# Posterolateral epidural supra-C2-root approach (PESCA) for biopsy of lesions of the odontoid process in same sitting after occipitocervical fixation and decompression—perioperative management and how to avoid vertebral artery injury

**DOI:** 10.1007/s10143-020-01468-z

**Published:** 2021-01-11

**Authors:** Patrick Haas, Till-Karsten Hauser, Kosmas Kandilaris, Sebastian Schenk, Marcos Tatagiba, Sasan Darius Adib

**Affiliations:** 1 Department of Neurosurgery, University of Tuebingen, Hoppe-Seyler-Str. 3, 72076 Tuebingen, Germany; 2 Department of Neuroradiology, University of Tuebingen, Hoppe-Seyler-Str. 3, 72076 Tuebingen, Germany; 3 Department of Neuropathology, University of Tuebingen, Hoppe-Seyler-Str. 3, 72076 Tuebingen, Germany; 4 Department of Anesthesiology and Intensive Care Medicine, University of Tuebingen, Hoppe-Seyler-Str. 3, 72076 Tuebingen, Germany

**Keywords:** Odontoid process, Vertebral artery, Posterolateral approach, Craniovertebral junction, 3D print, Occipitocervical fusion

## Abstract

This study aims to describe the posterolateral epidural supra-C2-root approach (PESCA), which might be a good alternative to the transoral, anterolateral, and other posterolateral approaches for biopsy of lesions of the odontoid process (OP). The preoperative planning of PESCA included computerized tomography (CT), CT-angiography, and three-dimensional reconstruction (if possible, even with three-dimensional print) to analyze the angle of the trajectory and the anatomy of the vertebral artery (VA). For PESCA, the patient is positioned under general anesthesia in prone position. In case of an osteolytic lesion with fracture of the OP, an X-ray is performed after positioning to verify anatomic alignment. In the first step, in case of instability and compression of the spinal cord, a craniocervical fusion and decompression is performed (laminectomy of the middle part of the C1 arc and removal of the lower part of the lateral C1 arc). The trajectory is immediately above the C2 root (and under the upper rest of the lateral part of C1 arc). Even if the trajectory is narrowed, it is possible to perform PESCA without relevant traction of the spinal cord. The vertical segment of V3 of the VA at the level of C2 is protected by the vertebral foramen, and the horizontal part of V3 is protected by the remnant upper lateral part of the C1 arc (in case of normal variants). PESCA might be a good choice for biopsy of selected lesions of the OP in same sitting procedure after craniocervical stabilization and decompression.

## Introduction

Surgeries of lesions of the odontoid process (OP) are still a great challenge today due to the close relation of the different important neurovascular structures.

Reddy et al. [25] concluded that usually three different approaches are used to C2 body and OP: (1) transoral approaches, (2) anterolateral, and (3) posterolateral (transpedicular) approaches.

The transoral-transpharyngeal approach provides access to the lower clivus, foramen magnum, anterior arc of C1, and the underlying odontoid process or C2, but the rate of infection and postoperative swelling using this approach is as high as 32 % [8, 13]; in addition, the surgical field is narrowed and deep [6], and there is high rate of CSF fistula.

Meanwhile the anterolateral and posterolateral transpedicular approaches are technically demanding due to the close relation with vascular structures (especially the V3 segment of vertebral artery (VA)) and neural structures, and the little size of the pedicles of C2 [25]. Apart from these main approaches, a good number of other approaches have been described in literature such as the lateral transcondylar approach [29], submandibular and transmandibular approaches [19, 30], the extreme lateral approach [29, 37], far lateral approach [11], and even open midline and posterior transdural approaches [1, 9]. There are also special percutaneous approaches for biopsy, kyphoplasty, and vertebroplasty of C2 (in particular anterolateral, transpedicular, transforaminal, paraspinal, posterolateral, translateral, far lateral [14, 27]) and special endoscopic approaches (in particular endonasal [26], transcervical [26], transoral [12, 26, 35], and far lateral [16]).

Al-Mefty et al. [20] recommended the transcondylar variant of open approach “with the removal of condylar surface of atlas for resection of the OP as an alternative to the anterior approach.” Türe et al. [37] demonstrated another posterolateral variant by transatlas access for the removal of the OP (extreme lateral-transatlas approach).

Riley et al. [26] were the first to recommend the METRx posterolateral approach, in which they did a small paravertebral incision and entered a METRx dilatator for a minimally invasive surgical approach for odontoid lesions.

Eissan and Eldin [6] showed that posterolateral approaches are associated with a high risk of VA injury, especially in case of bone drilling in this region. Geroge and Laurian [10] were the first to describe the techniques for the mobilization of VA. Eissa and Eldin [6] presented a new posterior midline approach that followed the same setting of occipitocervical fixation in a cadaver study (also with mobilization of VA). Even though interesting, they concluded that “one of the pitfalls and limitations of the study is the fact that many neurosurgeons still have a difficulty in exposure and mobilization of the vertebral artery even with this familial posterior approach.” As a result, we could not find any clinical report about this approach in real patients. Is there a save corridor without direct contact to VA?

Our objective was then to find an approach that follows the same sitting of posterior stabilization and decompression to get to the odontoid process for biopsy of unclear lesions, without opening the dura and without contact with the vertebral artery.

Therefore, we want to present the posterolateral epidural supra-C2-root approach (PESCA).

## Methods

### Case presentation

**Fig. 1:**
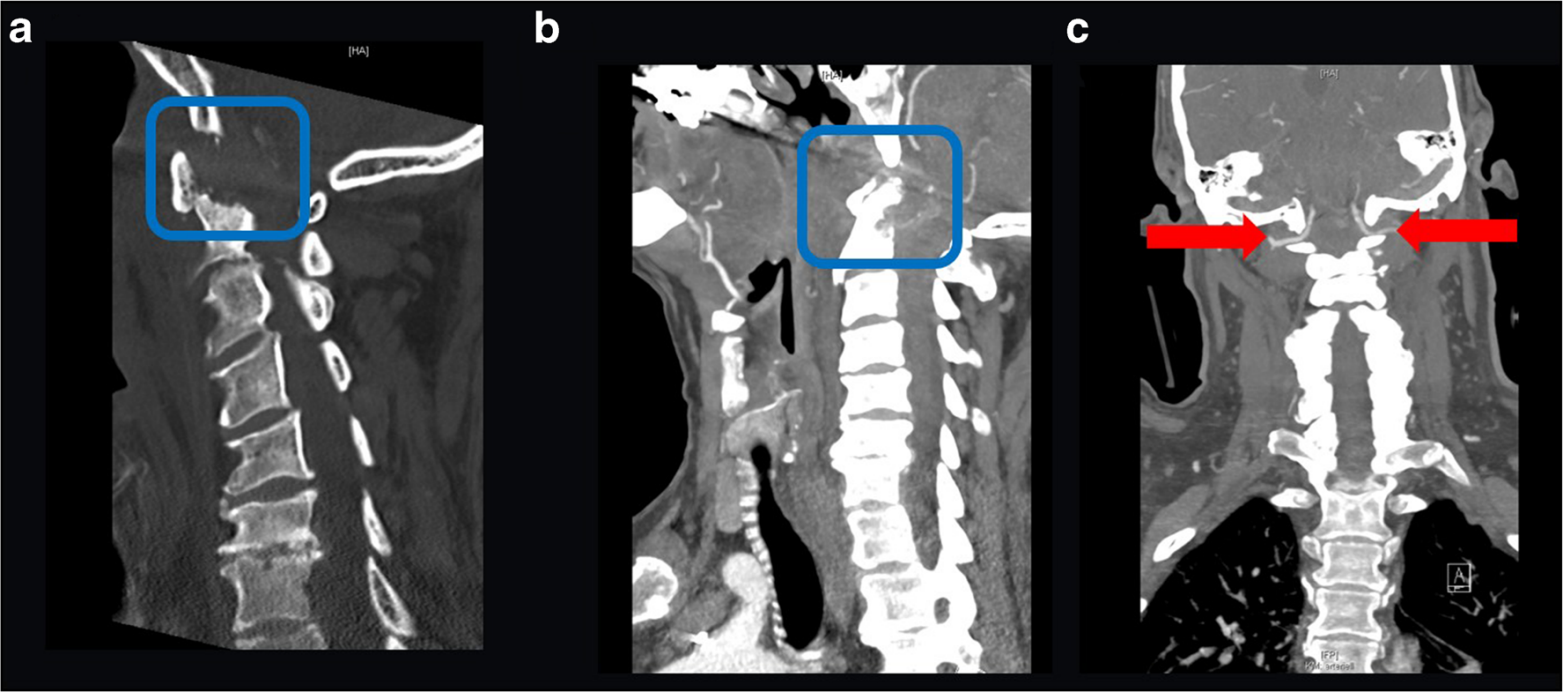
Preoperative CT and CTA of cervical spine: **a** sagittal CT (bone window) revealed an osteolytic lesion of the OP (blue frame); **b** sagittal CT (soft tissue window) showed compression of spinal cord (blue frame); **c** coronal CT revealed “normal” variant of V3 segment of VA (VA runs over the posterior arch of C1) (red arrows)

A 72-year-old man presented with a 4-week history of headache and neck pain, with a weight loss of over 20 kg in 6 months. A computed tomography (CT) of the craniovertebral junction (CVJ) and the cervical spine was done, which revealed an osteolytic lesion of the odontoid with signs of instability (differential diagnosis (DD) metastasis, DD rheumatoid arthritis, DD spondylodiscitis) (Fig. 1 a and b). MRI was not possible due to the presence of a pacemaker.

As history, the patient had a mitral valve insufficiency and coronary heart disease, with stent implantation 11 months ago (the patient takes clopidogrel), had an arterial fibrillation with a pacemaker, was hypertensive, and had a renal insufficiency.

Firstly, he was given a Philadelphia collar and analgesics.

Because the patient had severe secondary diseases, we had to find a strategy to solve these problems in one surgery.

An approach through midline incision from posterolateral which can be done in same sitting during posterior craniocervical stabilization was necessary.

### Goals of surgery

1. To prevent any new perioperative problem (thrombosis, bleeding, stent occlusion, new neurological symptoms)
2. Posterior stabilization of the CVJ (C0–C4: OC plate, pedicle screw C2, and mass laterals screw C3 and C4) due to instability with the risk of neurological impairment
3. Laminectomy C1 and enlargement of the foramen magnum for decompression
4. Biopsy of the odontoid lesion to ensure the correct diagnosis
5. Single session for all procedures (due to severe secondary diseases and additional risk of a second surgery)

### Reasons for PESCA

We thought of different possibilities to attain our objectives with only one surgery. To attain the second and third objectives, a prone position and a posterior approach was needed. In order to attain all our objectives in one procedure (goal 5), we had to find a way to do a biopsy because of the posterior approach (goal 3). It was not possible to reach the lesion through a posterior lateral transpedicular approach (because it was not possible to reach the osteolytic part of the OP through the angle of the pedicle); thus, we looked for other possibilities.

In the case of the posterolateral approaches to the OP the most “dangerous” structures are the VA and the spinal cord. We had to find a safe trajectory as regards these two structures.

Because of the serious secondary diagnoses, we planned a three-step surgery with the possibility of stopping at any step (even with partial attainment of the objective) depending on the occurrence of potential problems such as blood loss, cardiac, or pulmonary problems.

### Preoperative planning of strategy

A thin sliced CT scan of the cervical spine for spinal neuronavigation and virtual 3-dimensional reconstruction was done.

A CT angiography (CTA) for analysis of the V3 segment of VA was done which revealed a “normal” anatomy of V3 segment (Fig. 1c).

Based on the thin-layer CT data set, a digital volume rendering model was generated. For preoperative planning of the safe biopsy corridor and simulation of the individual surgical steps, this data set was produced as a 1:1 scale model using the Fused Filament Fabrication (FFF, also known as 3D printing). The thermoplastic polyester polylactic acid (PLA) was used as a printing substance (filament).

The goal was to find the “safe” corridor. Different corridors were tried in the 3-dimensional model before surgery. A complex anesthesiological management was performed with normalization of coagulation.

## Surgical technique/surgical steps

### Intervention

The patient was positioned in a prone position under general anesthesia (head in a straight position, without rotation or inclination, due to instability and the risk of spinal cord injury) in accordance to our standards. Cefuroxime was administered for perioperative surgical prophylaxis. An X-ray was done after positioning to verify anatomic alignment.

Intraoperative monitoring included sensory evoked potentials (SWP) and motor evoked potentials (MEP) of the upper and lower extremities.

We did a midline incision because posterior fusion and decompression were also done. A monopolar electrocautery could not be done due to the presence of the pacemaker. The inion, posterior wall of the posterior cranial fossa, C1 arc, and laminae C2, C3, and C4 were exposed.

#### Step 1: fusion of the craniocervical junction C0–C4

A C0 to C4 fusion was done in the first step with an OC plate (DePuy: Mountaineer). The pedicle screw C2 was placed under spinal neuronavigation (Brainlab) and at the level of C3, and C4 massa lateralis screws were inserted.

#### Step 2: decompression

**Fig. 2:**
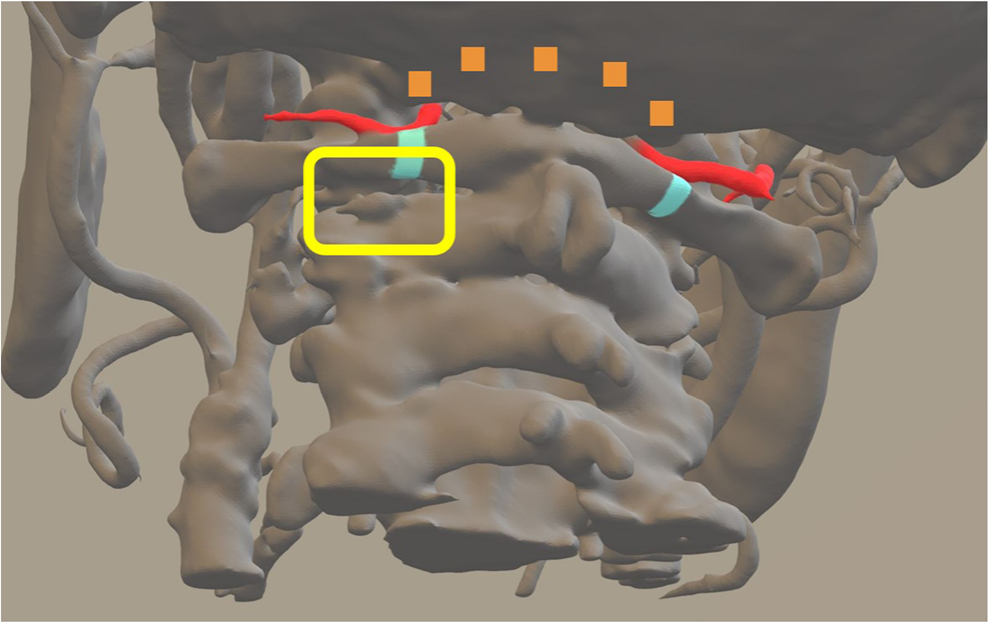
3-Dimensional reconstruction of the cervical spine: red structures = vertebral arteries; orange squares = enlargement of foramen of magnum; light blue squares = laminectomy of the medial part of C1, yellow frame = entry zone for PESCA

An enlargement of foramen magnum was performed under the microscope (Carl Zeiss: kinevo) and a laminectomy of the medial C1 arc and the lower lateral part of C1 arc (subperiostal, with remnant upper C1 arc), and the removal of the left superior part of the left side of the arc of C2 and flavectomy was done (the distance between lateral upper C1 arc and inferior left part of C2 arc was around 1.5 cm) (Fig. 2).

#### Step 3: biopsy of the lesion of the odontoid process through PESCA

Various landmarks such as C2 root, the remains of C1 arc, C2 arc, and the dural sac were identified. With the help of spinal neuronavigation, we checked our trajectory (especially the viewing and the working angle).

**Fig. 3:**
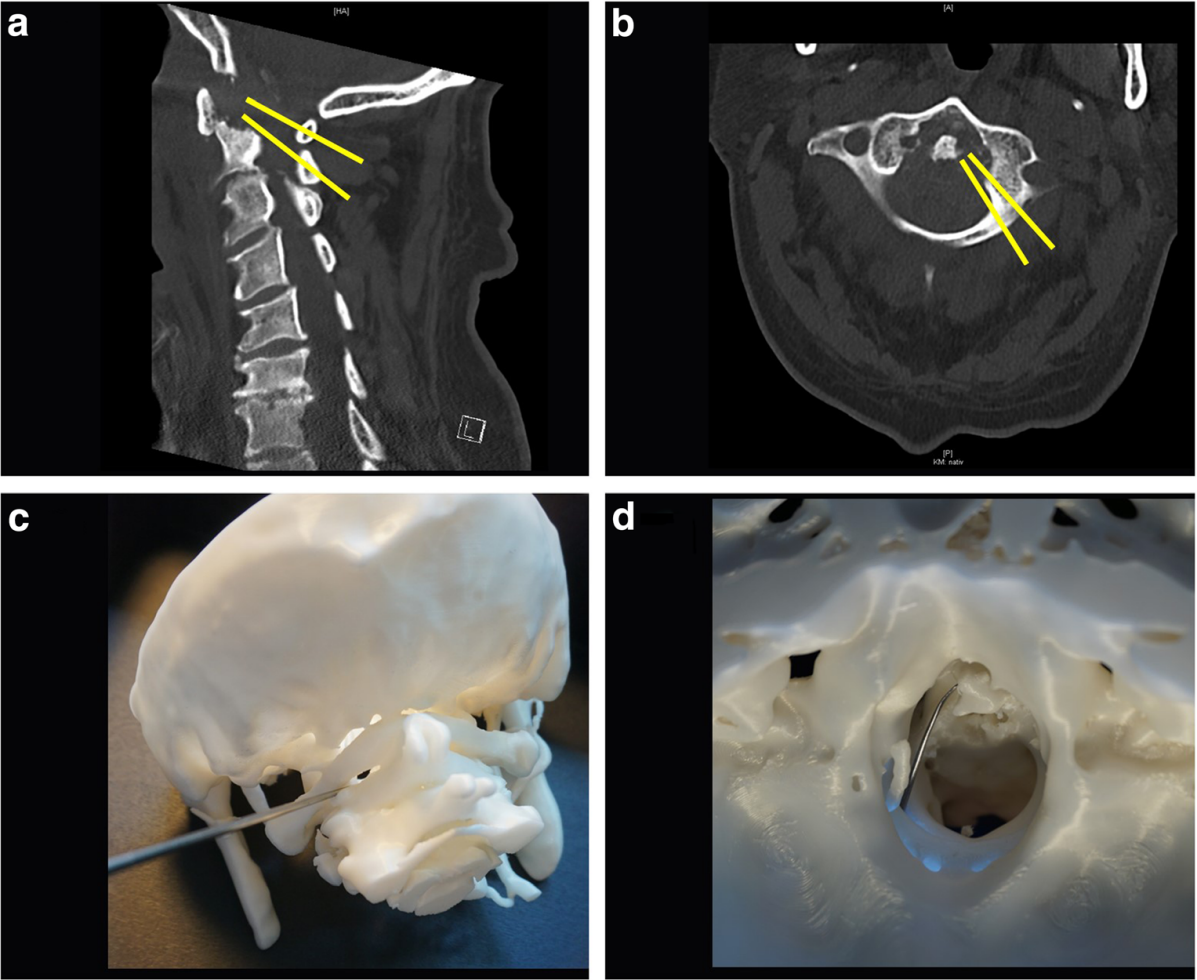
**a** Sagittal CT (bone window): yellow lines = trajectory of PESCA. **b** Axial CT (bone window): yellow line = trajectory of PESCA. **c** 3-Dimensional printing of the cervical spine (the inserted dissector demonstrates the entry point). **d** 3-Dimensional printing of the cervical spine: view of OP and foramen magnum (the inserted dissector demonstrates that PESCA is possible without relevant traction of the spinal cord)

As close as possible to the superior part of C2 root, a dissector was carefully inserted with slight traction of the dural sac (under microscope and under IOM). The window between the rest of C2 arc, and C2 root was used as our approach (Fig. 3). Directly above the superior part of the C2 root was the entry point. The trajectory was located medial to pedicle of C2, medial to C1–C2 facet joint, and medial to tubercle for transverse ligament of atlas.

Using the left hand, the trajectory was slightly enlarged with a dissector by a slight touch to the dura, and with the right hand, a bioptic instrument was inserted under the microscope. The space needed had to be enough for just for one microinstrument. Due to dorsal decompression, the danger of compression was limited as much as possible. The IOM did not show any changes in MEP or SEP.

The OP was then exposed. A biopsy of the lateral portions of the lesions was done. The first intraoperative pathological analysis did not show a clear result; therefore, a second probe from the odontoid process was done, which revealed a spondylodiscitis without any signs of tumor or rheumatoid disease. After biopsy, the rest of C1 arc was removed. IOM remained stable during whole surgery.

### Postoperative course

**Fig. 4:**
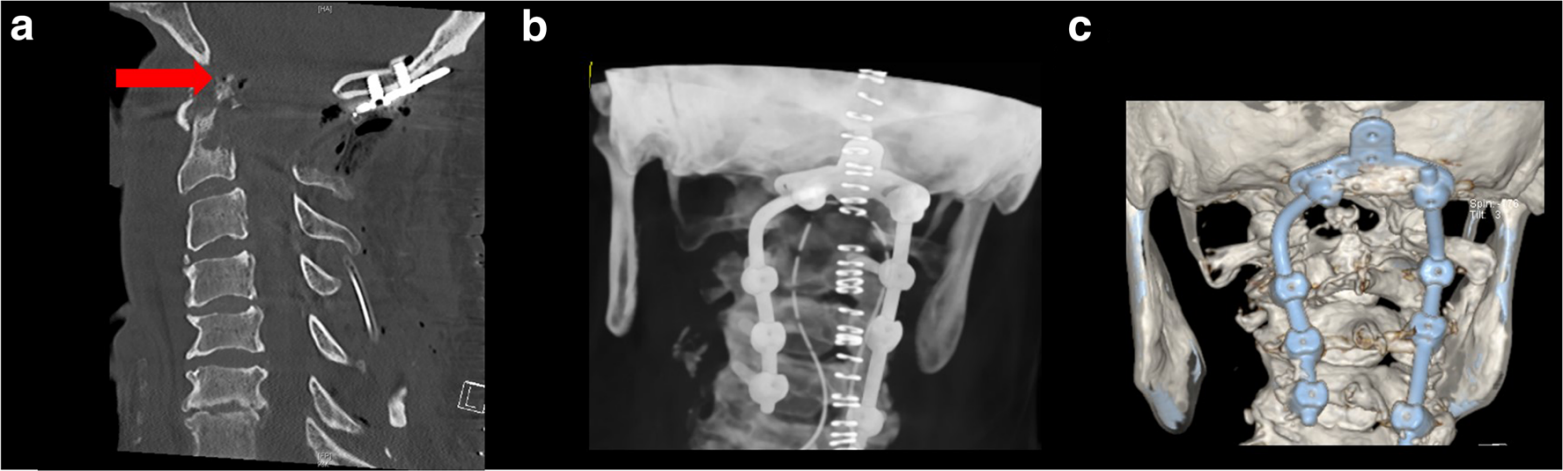
Postoperative CT scan of cervical spine (**a**) and different 3-dimensional reconstruction (**b,c**) revealed a regular placement of screws and air in the OP from biopsy (red arrow)

The patient recovered from surgery without any new deficits, but still with head and neck pain. A postoperative CT scan revealed a proper positioning of the screws and sufficient decompression of the spinal cord at the level of the CVJ (Fig. 4).

**Fig. 5:**
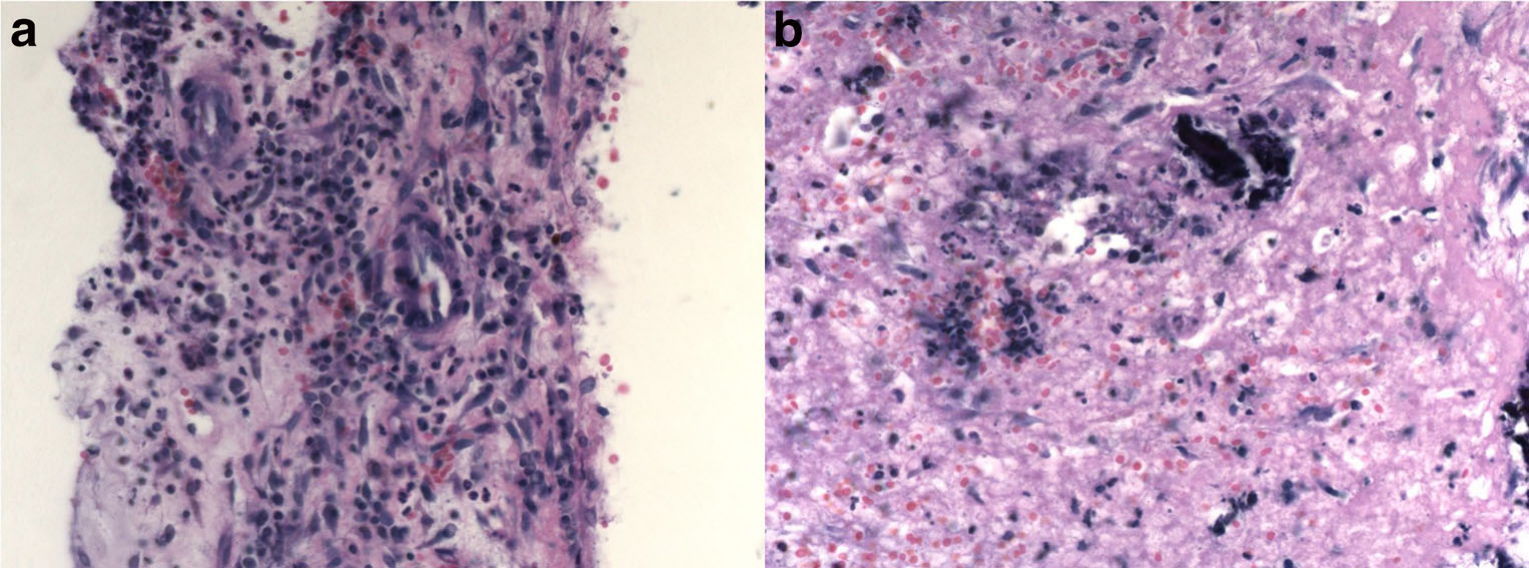
**a,b** Histopathologic findings of bioptic probe (hematoxylin-eosin stain), showing inflammatory tissue with neutrophil granulocytes, plasmocytes, and lymphocytes

Pathologic examination of the mass showed a chronic recurrent spondylodiscitis without any signs of tumor (Fig. 5). An empiric antibiotherapy with clindamycin and ceftriaxone was started.

After a few days, a wound revision was performed due to wound infection with screw loosening in the occipital plate, and screws with larger diameter were inserted.

Microbiological analysis of the probe and sore smears during the second surgery revealed *Corynebacterium striatum* with multiple resistance (even with resistance to clindamycin). Wound infection was caused by insufficient empiric antibiotherapy due to varying resistances of the very rare bacteria (Corynebacterium striatum) which was responsible for the spondylodiscitis of the OP and the secondary wound infection.

Retrospectively, the patient had a diabetic foot with wound infection one year ago, in addition to a number of other infections. Microbiological probes of the diabetic foot had also revealed *Corynebacterium striatum* with the same resistance; therefore, this seems to be the focus of spondylodiscitis of the OP.

These findings were the key to the successful treatment of the disease. The antibiotherapy was changed to vancomycine. Ten days after the initiation of vancomycine, the patient was transferred in a good condition with decreased infection parameters to his home hospital.

### Follow-up

**Fig. 6:**
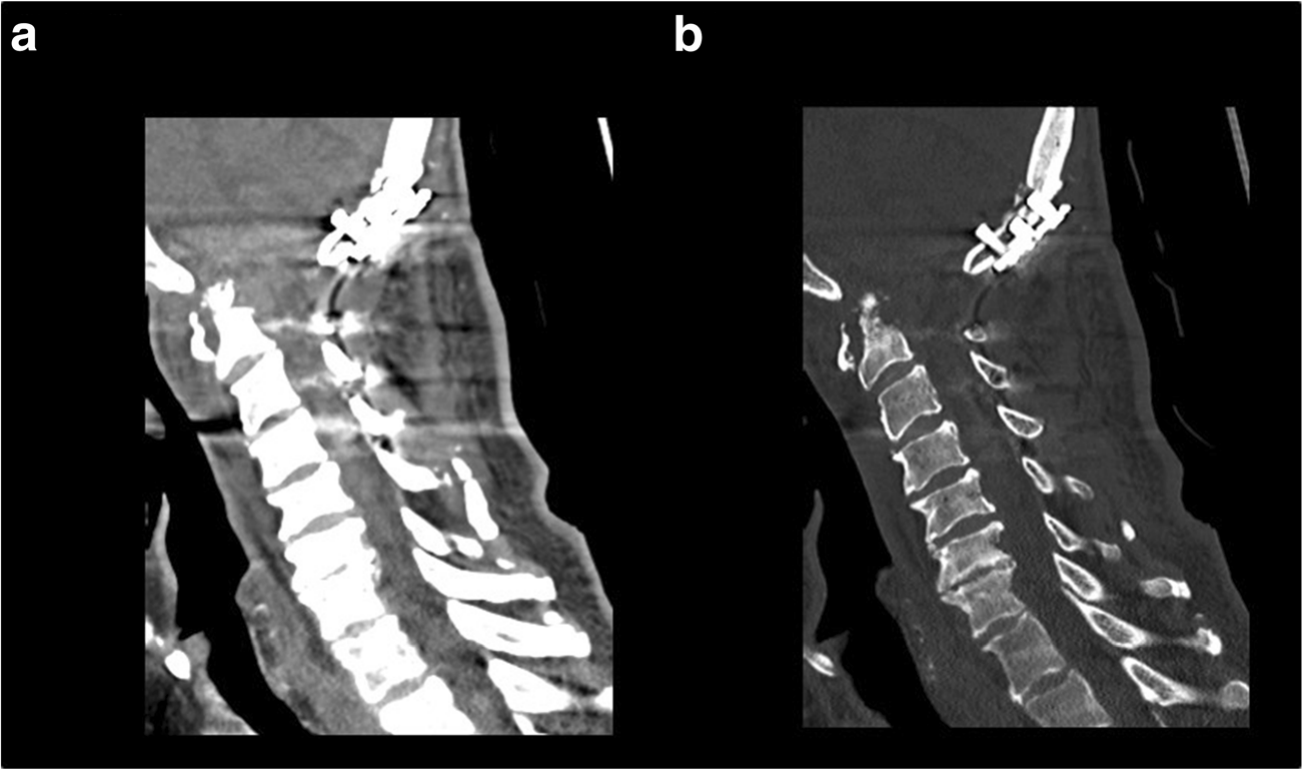
Sagittal CT scan of the cervical spine (**a** soft tissue window; **b** bone window) revealed no postinfectional changes of the OP without further destruction of the OP and revealed no signs of screw loosening

At follow-up examination 1 month after surgery, the patient did not manifest any neurological symptoms or symptoms of infection. The C-reactive protein level was 1.28 mg/dl (versus 11.62 mg/dl at 1 month ago). Wound healing was uneventful. A CT scan of the cervical spine revealed postinfectional changes of the OP without further destruction of the OP, and there were no signs of screw loosening (Fig. 6 a and b).

## Discussion

Reddy et al. [25] concluded that “lesions involving the dens and body of C2 are unique as it is quite challenging to approach these lesions for surgery or other interventions due to the close proximity to several important neural and vascular structures.”

The study aimed to find a safe corridor to the OP for biopsy of indistinct lesions from the posterolateral approach, which can be used in the same setting as posterior stabilization and decompression, without opening of dura. Therefore, we developed the “posterolateral epidural supra-C2-root approach” (PESCA). For PESCA, we performed a medial C1 arc removal with drilling of the lower part of the lateral C1 arc (subperiostal, with remnant upper C1 arc) and C2 partial arc removal. The window between the rest of the C2 arc and the C2 root was used in our approach.

### Osteolytic lesions of the OP

Rheumatoid arthritis is the main pathology which leads to osteolysis of the odontoid (approximately 58% to 72% of the cases). Spondylodiscitis of the odontoid is rare [4], but according to literature, the mortality rate is approximately one-third [17, 26, 33]. Tuberculosis is the most common infection in the atlantoaxial region [4].

The most nonspecific symptoms (such as neck pain, fever, and swelling of the neck) are common [4]. In our case, microbiological analysis of the probe and sore smears revealed the presence of Corynebacterium striatum with varying resistances (even resistant to clindamycin). A Corynebacterium infection of the OP is very rare.

### V3 segment of vertebral artery: anatomy, variants, and injury in cervical spine surgery

One main objective was to search for an approach with a safe distance to VA (or even without contact to vertebral artery) to prevent VA injury. Therefore, the study of V3 segment of VA and its variants was an important part. The V3 segment of VA is located at the level of the craniocervical junction (CVJ) (from C-2 transverse process to the artery’s entry through to the dura mater [39]) is anatomical complex [39, 40] and can be subdivided into 3 parts [10, 39].

Wakao et al. [40] concluded that VA injury “is one of the most serious complications arising from cervical spine procedures.” The rate of VA injury during cervical spine surgery ranges from 0.3 to 8.2% in normal anatomy [15, 18, 21, 22].

Injury to the dominant VA (but sometimes also the nondominant VA) may result in severe complications such as a pseudoaneurysm, a formation of an arteriovenous fistula, VA occlusion, severe bleeding, stroke, or even death [39].

Different variants of the VA have been reported by different authors [28, 32, 36, 41, 42], and Ulm et al. [39] analyzed the different distances.

Wakao et al. [40] classified the different variants of the VA in the CVJ into (1) persistent first intersegmental artery (FIA) (1.8%), (2) fenestration of the VA above and below C1 (FEN) (1.3%), (3) posterior and inferior cerebellar artery (PICA) from C1/2 (1.3%), and (4) high-riding VA (10.1 %). Li et al. [18] recommended a different classification with seven different types of variation of the VA at the CVJ. Tokuda et al. [36] and Yamazaki et al. [42] found a relationship between VA anomalies and anomalies of the CVJ (e.g., in case of Klippel–Feil syndrome).

In case of FIA and in case of FEN, PESCA is not a good choice. Patra et al. [24] showed that embryologically, the VA is formed by vertical channel interconnecting the cervical intersegmental arteries. Intersegmental arteries disappear while the vertical connections persist [22, 24].

Tokuda et al. [36] also showed in their series of 300 vertebral artery that in two cases, the VA runs under the posterior arch of C1 [31, 36], and Uchino et al. [38] reported a persistence of first intersegmental artery in up to 3.2% of normal subjects and an overall prevalence of CVJ-VA anomalies of 5%. Before carrying out the PESCA, CTA of the vertebral artery is absolutely necessary.

In these cases (FIA and FEN), PESCA should not be done because of the high risk of VA injury.

### Advantages of the 3-dimensional printing

Additive manufacturing, also known as 3D printing, is a rapidly emerging technology in the medical sector. Besides the production of patient-individual implants, patient education, or university teaching, 3D-printed models enable the detailed simulation of an upcoming operation or serve the development of new operation methods. Even they cannot replace an anatomical preparation, they offer the advantage of reproducing the individual site exactly and without great expenditure of time and money. Thermoplastic Fused Filament Fabrication (FFF) and photochemical polymerization using stereolithography (SLA, resin printing) have proven themselves for the production of anatomical models. They are available as desktop printers in a variety of different makes at low cost. More sophisticated processes such as selective laser sintering, on the other hand, require a much more complex infrastructure and are more reserved for applications such as the development of implants.

3D-printed models are not to be understood as competition to the purely digitally generated models. Rather, they are the logical continuation of digitization and thus enable the technological bridging back into a haptically oriented discipline such as neurosurgery.

The idea of PESCA was created using this 3-dimensional print and analyzing different potential corridors.

However, even if helpful, not every hospital has the capacity to perform 3D printing. With the abovementioned anatomical and radiological knowledge, PESCA can also be performed without preoperative 3D-printed model.

### Indications for PESCA

PESCA provides a safe corridor. This approach is safe regarding the vertebral artery in normal constellation. At the level of C2 the vertebral artery is normally covered by bone and clearly away from the approach. The horizontal segment of V3 normally turns around the C1 arc. This means that under the C1 arch, there is a safe entry zone.

In case of a necessary posterior fusion of CVJ with decompression with laminectomy of C1 (and partial resection of lamina C2, and possibly decompression of foramen magnum) due to an osteolytic lesion of the OP with instability, the PESCA can be a good choice, especially in patients with severe secondary diagnoses with the goal of only one surgery for fusion, decompression, and bioptic procedure.

The main advantage of the PESCA is that no secondary approach is needed (compared with transoral or anterolateral approaches). Furthermore, in the transoral approach, the rate of infection and postoperative swelling using this approach reaches up to 32% [8, 13]; in addition, the surgical field is narrowed and deep [6], and there is a high rate of CSF fistula. Moreover, the access may be limited by anatomical problems like plate size and location [26], and in many cases, an ENT co-surgeon is necessary [26]. In case of posterolateral transpedicular approaches, owing to the trajectory (because of the angle of the pedicle), the reachable target is limited, and it might be difficult to reach the upper parts of the OP. A further advantage of the PESCA, compared with the posterolateral transpedicular approaches, is that the surgeon works under a view.

Posterior transdural approaches are associated with a high risk for cerebrospinal fluid leakage and infections [26], and a previous study reported a risk of accessory nerve injury or vertebral artery injury [26].

Two structures should be kept in mind: (1) The dura sac with spinal cord and (2) the vertebral artery. Intraoperative monitoring with MEP and SEP might be of benefit to get an overview of the spinal cord function. In case of deterioration of MEP or SEP, the approach should be discontinued and another one decided upon.

A very careful planning of the trajectory should be done to make sure that the lesion of OP can be reached through PESCA.

A strong knowledge of the course of VA is absolutely necessary, especially with VA variations as focus (see below).

Different authors [22, 39] reported that the risk of intraoperative vertebral injury during surgeries of the CVJ can be reduced with the aid of preoperative imaging that included the evaluation of osseous cervical structures and vascular anatomy [39].

We recommend that CT with CTA and, if possible, MRI should be performed. In addition, 3-dimensional virtual reconstructions and/or 3-dimensional prints provide a major advantage in preoperative planning.

In very special anatomical constellation, a sonographic Doppler helps for a safe surgery.

### Enlargement of the posterolateral pathway to the OP: head positioning, sacrification of C2 (and/or C1)

In our case, we did a medial C1 arc removal with drilling of the lower part of the lateral C1 arc, C2 partial arc removal, and craniotomy for enlargement of foramen of magnum [30].

Positioning of the head might help to enlarge the anatomical window [26]. In our case, the odontoid was osteolytic, and consequently we positioned the head in straight position to prevent fracture of odontoid process with injury of spinal cord, furthermore, to be in right position for stabilization.

Depending on the anatomy, an infra-C2-root approach (instead of a supra-C2-root approach) might be discussed.

Even PESCA is a supra-C2-root approach, in case of a narrowed corridor of PESCA, a sacrifice of C2 root might be discussed, because mobilization of the C2 root can be difficult even for the most experienced surgeons [34].

But, sacrificing the C2 root should not be done generously, due to the fact that it might result in occipital hypesthesia [7] and in up to 25% of cases in neuropathic pain syndrome [3, 7]. However, Badhiwala et al. [3] summarized that there is a substantial heterogeneity in the outcomes of numbness and occipital pain after sacrifice of the C2 root in the literature [3]. They concluded that the studies are different regarding design, method of assessment of C2 function, and primary outcome [3]. Also, different techniques of C2 sacrification had been described, in case of C 1 lateral mass screws, in literature [2, 7, 23], which might improve exposing of relevant anatomy. Besides monopolar [21, 34] and bipolar electrocautery [2, 43], also sharp division [23] has been described. Ligating the cut nerve ends has been reported [5]. Florman et al. [7] concluded that review of publications addressing the occurrence of postoperative neuralgia after C2 root division also reveals substantial variability in sectioning practice and reported that C2 neuralgia is rare in case of sharply dividing the C2 root with bipolar electrocautery [7]. Furthermore, the location of the C2 transection seems to be relevant [7]. C2 neuralgia seems to be rarer in case of dividing C2 root at the midportion of the ganglion where it overlies the C1–2 joint [7]. Furthermore, hemorrhage of the venous plexus during the procedure has been described as a potential risk [34].

In case of stabilization, also C2 arc removal could be discussed. In case of narrowed corridor using PESCA, drilling of lateral mass and/or mobilization of vertebral artery [6] would help to enlarge the pathway.

Riley et al. [26] were able to safely access the anterior epidural space, odontoid, and even retropharynx. In their approach, they passed between thecal sac and the C1/2 joint, but they used an approach 4 cm lateral to the midline and enter a METRc (Medtronic, Memphis, TN) dilatator. The C1 inferior laminar edge was shaved down by 2–3 mm [26].

### Limitations

In our opinion, PESCA is an approach used especially for biopsy of odontoid lesions, due to the fact that the corridor is very narrowed and the working angle is orientated up.

Removal of odontoid process is not possible. Furthermore, it is not suitable for anterior lesions of the OP.

In case of special constellation of anatomy of VA such as intersegmental VA, PESCA is contraindicated.

Due to the fact that slight traction of dural sac maybe necessary, PESCA should be only performed after previous decompression (enlargement of foramen of magnum and medial C1 laminectomy with drilling of the lower lateral C1 arc is furthermore necessary, and even partial removal of C2 arc), and IOM is absolutely necessary.

In case of small C1 and C2 arc with close relation to each other, PESCA is not a good choice.

## Summary

In summary, PESCA is a good approach for biopsy of lesions of the OP after CVJ fusion and laminectomy or subperiostal laminectomy of C1 and possibly partial removal of one side of C2 arc (and eventual scarification of root C2). Even a second surgery was necessary in our case because of a wound infection (due to insufficient empiric antibiotherapy before final microbiological analysis); we demonstrated that PESCA is possible in the same sitting after occipitocervical fixation and decompression.

The huge advantage of PESCA is that there is no direct contact to VA in case of “normal variant of VA” and in case of subperiostal C1 removal.

It is absolutely necessary to carry out a careful preoperative planning with CTA of vertebral artery; in addition, a 3-dimensional print may help to get familiar with the individual anatomic situation. Even 3-dimensional virtual reconstructions are common today; the haptic printing will help the trajectory in a different way. Also, it may help to study the working angle to the OP. IOM is essential, and spinal neuronavigation will help to find the best trajectory. In a very special anatomical constellation, a sonographic Doppler might also support safe surgery.

A good team with experienced anesthetists and spinal surgeon is necessary.

It is important to recognize that this approach has a small corridor and a steep angle, but it is elegant, due to the fact, that you do not have to use a second approach after fusion and decompression.

## Data Availability

All data are included in the manuscript.
